# The potential role of spectrin network in the mechanotransduction of MLO-Y4 osteocytes

**DOI:** 10.1038/srep40940

**Published:** 2017-01-23

**Authors:** Xin-Tong Wu, Lian-Wen Sun, Xiao Yang, Dong Ding, Dong Han, Yu-Bo Fan

**Affiliations:** 1 Key Laboratory for Biomechanics and Mechanobiology of Ministry of Education, School of Biological Science and Medical Engineering, Beihang University, 37^th^ Xue-yuan Road, Hian-dian District, Beijing, China; 2 International Joint Research Center of Aerospace Biotechnology and Medical Engineering, Ministry of Science and Technology of China, School of Biological Science and Medical Engineering, Beihang University, 37th Xue-yuan Road, Hian-dian District, Beijing, China; 3 National Center for Nanoscience and Technology, Beijing, China; 4 National Research Center for Rehabilitation Technical Aids, 1^th^ Ronghuazhong Road, Beijing Economic and Technological Development Zone, China

## Abstract

The spectrin is first identified as the main component of erythrocyte membrane skeleton. It is getting growing attention since being found in multiple nonerythroid cells, providing complex mechanical properties and signal interface under the cell membrane. Recent genomics studies have revealed that the spectrin is highly relevant to bone disorders. However, in osteocytes, the important mechanosensors in bone, the role of spectrin is poorly understood. In this research, the role of spectrin in the mechanotransduction of MLO-Y4 osteocytes was studied. Immunofluorescence staining showed that, the spectrins were elaborately organized as a porous network throughout the cytoplasm, and linked with F-actin into a dense layer underlying the cell membrane. AFM results indicate that, the spectrin is pivotal for maintaining the overall elasticity of osteocytes, especially for the cell cortex stiffiness. Disruption of the spectrin network caused obvious softening of osteocytes, and resulted in a significant increase of Ca^2+^ influx, NO secretion, cell-cell connections and also induced a translocation of eNOS from membrane to cytoplasm. These results indicate that the spectrin network is a global structural support for osteocytes involving in the mechanotransduction process, making it a potential therapeutic target for bone disorders.

Osteocytes are acknowledged as the “mechanosensors” in bone. Mechanical stimuli upon osteocytes can be converted to bio-chemical signals and propagate within the osteocytes network, which is called the mechanotransduction of osteocytes^1^. Previous studies have demonstrated that, the ECM (extracellular matrix), the membrane receptors (including the integrin, caveolin and lipid raft), the cytoskeleton and the cell nucleus are all acting as the key elements in mechanotransduction from extracellular to intracellular^2^,^3^. In addition to these structures, the spectrins located between the plasma membrane and the nucleus, first identified as a structural component of membrane skeleton of erythrocyte^4^, are also found to be involved in the cytoskeleton system of multiple nonerythroid cells, including bone cells^5^,^6^. Together with F-actin, ankyrin and protein 4.1 etc, the spectrins form a scaffold throughout the cytoplasm^7^. On one hand, the spectrins are targeted to the cell membrane through the PH domain^8^, and on the other hand they are directly associated with F-actin and microtubules^9^. Besides its structural function in cells, the spectrin network is also demonstrated to be an important interface for numerous signal pathways, and involved in multiple physiological processes of nonerythroid cells^10^,^11^,^12^.

Recently, growing evidence has shown that the spectrins are of great relevance to bone disorders. Genotyping studies have revealed that the SPTBN1, a member of the β-spectrin gene family, is one of the 20 BMD (bone mineral density) associated locis, and is highly related with the bone fracture risk^13^,^14^,^15^,^16^. However, in osteocytes, the important mechanosensors in bone, the role of spectrins is infrequently studied.

It is generally acknowledged that, the mechanical properties of cell are closely related to the cellular mechanosensitivity^17^. Upon mechanical stimulations, a certain value of strain or deformation of the plasma membrane is necessary to activate the membrane-associated receptors and trigger the downstream biochemical responses^18^. The cellular deformation after mechanical stimulations is determined by the elasticity or stiffiness of cells, which is dominated by the tension of the cytoskeleton^19^. While the spectrin, organized as a flexible platform underneath the cell membrane also greatly contributes to the mechanical properties of cells^20^. However, the effect of spectrin on the mechanical properties of osteocytes has not been covered before.

On the other hand, a series of biochemical responses of osteocytes will be initiated upon mechanical stimulations, including the secretion of NO (nitric oxide) and Ca^2+^ influx. The mechanical signals will be spread to adjacent osteocytes through gap junctions^21^. In the “window washer” model proposed by Jenkins^22^, it is suggested that the spectrin-ankyrin network is like an active patrol of cell membrane to inhibit endocytosis. So it is speculated that, the transmembrane Ca^2+^ channel, eNOS and the cell connexins should also be within the “jurisdiction” of spectrin network, but the regulation of spectrin on these mechanosensitive indexes in osteocytes has not been studied.

Therefore, the spectrin of MLO-Y4 osteocytes was explored in this study. The distribution of spectrin and the relative location between the spectrin network and F-actin microfilament were studied. The contribution of spectrin network to cell stiffiness was measured by AFM (Atomic Force Microscope). Also, the effect of spectrin network on NO and intracellular Ca^2+^ signal, as well as the formation of osteocyte-osteocyte connections were studied to reveal the role of spectrin in the mechanotransduction of MLO-Y4 osteocytes.

## Results

### Distribution of the spectrin in MLO-Y4 and the effect of DIA on spectrin network

**Figure 1:**
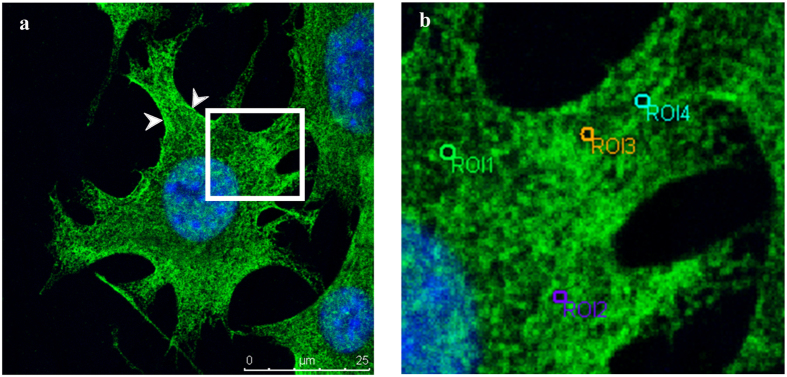
The spectrin of MLO-Y4 was fluorescence stained with Alexa 488 (green) and the nucleus was labeled by DAPI (blue) to locate cells. The spectrin was observed on the cell membrane, and also in the cytoplasm and nucleus. Some intense stainings were found on the membrane (indicate by arrow heads) (**a**). The box inset in (**a**) was amplified (**b**), and it showed that the spectrin was organized to a porous network structure. The grid of the spectrin network was selected as a region of interest (ROI) for measurements.

The distribution of spectrin in MLO-Y4 cells was examined. Results showed that the spectrin is widely distributed in osteocytes. It is observed not only on the cell membrane, but also throughout the cytoplasm, and even in the nuclei (Fig. 1a). Under high magnification, it can be shown that the spectrin is organized as a reticulate network with opening holes (Fig. 1b). The circular selection tool of the image processing software (Leica Application Suite) was adopted to select the grid of the network (the circle in Fig. 1b) for quantification measurement. Results showed that the diameter of the grid was 0.73 ± 0.29 um and the area was 0.35 ± 0.02 um^2^ (Table 1).

**Figure 2:**
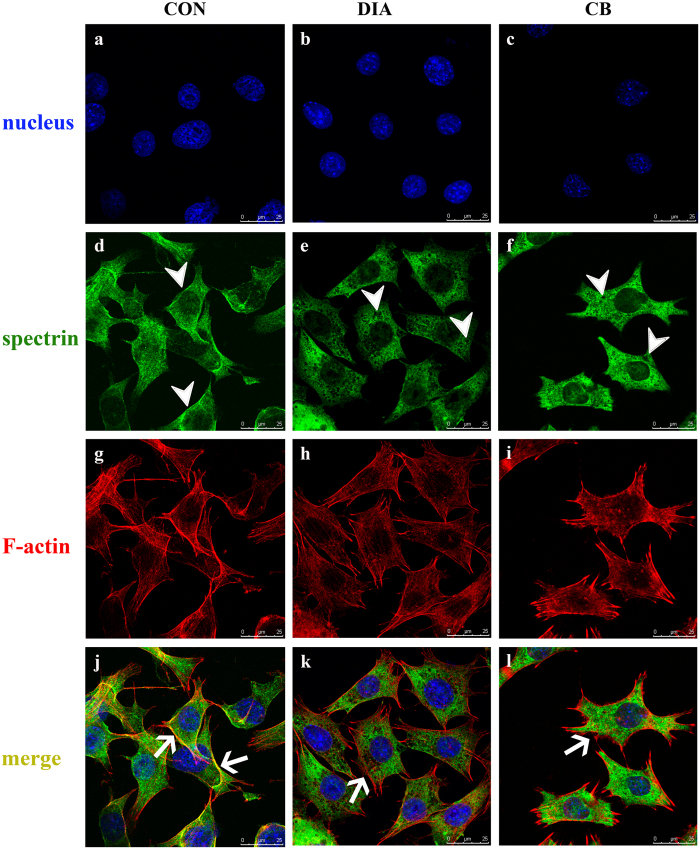
The distributions of spectrin and F-actin of osteocytes were examined by the double-label fluorescence staining. In the control group (**j**), the spectrin network (green) was co-localized with the F-actin (red) on the membrane (golden, indicated by the arrow); the treatment of DIA disrupted the spectrin network, and some holes were shown in the cytoplasm (**e**, indicated by the arrow head), while the F-actin stress fibers were unbroken (**h**), but the colocalization of spectrin and F-actin on the membrane disappeared (**k**, indicated by the arrow); the treatment of CB also broke the spectrin network (**f**, indicated by the arrow head), but to a lower degree compared with the DIA group, and the F-actin was disrupted to short and broken fibers (**i**), and the colocalization with spectrin on the membrane also disappeared (**l**, indicated by the arrow).

The diamide (DIA) and cytochalasin B (CB) were used to disrupt the spectrin and F-actin network respectively. MTT assay results showed the treatment of DIA or CB did not affect the survival rate of MLO-Y4 osteocytes (data not show). In untreated osteocytes, the F-actin and spectrin were co-localized on the cell membrane with intense gold staining, but the co-localization is not observed in the cytoplasm and nucleus (Fig. 2j). After the treatment of DIA, the structure of the spectrin network was disrupted with big holes within the network (Fig. 2e), and the co-localization with F-actin on the membrane disappeared (Fig. 2k). The F-actin stress fibers were undamaged by DIA (Fig. 2h). The treatment of CB broke the F-actin stress fibers as reported^23^ (Fig. 2i), and the spectrin network were also damaged, but in a lesser extent compared with the DIA group (Fig. 2f). The co-localization of spectrin and F-actin on the cell membrane also disappeared (Fig. 2l). The diameters of the spectrin grids after drug treatments were measured, and the results showed that the treatment of DIA or CB both significantly increased the diameter and area of the spectrin grid compared with CON (Table 1).

### Effect of the spectrin network on Young’s modulus of MLO-Y4

**Figure 3:**
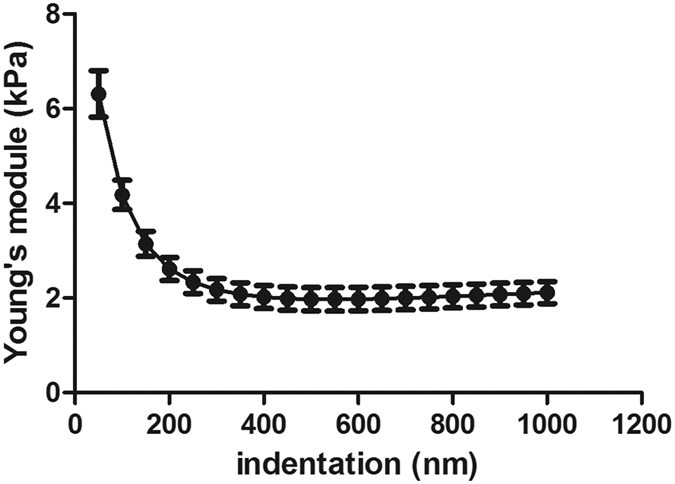
The Young’s modulus of MLO-Y4 was measured by AFM. At different indentation depth, the cell stiffiness varies greatly, and with the indentations getting deeper, the Young’s modulus gets smaller. At cell cortex, the Young’s modulus was 6.31 ± 0.49 kPa, 4.18 ± 0.31 kPa, 3.15 ± 0.27 kPa, 2.62 ± 0.24 kPa respectively at 50 nm, 100 nm, 150 nm and 200 nm. At 500 nm, the Young’s modulus decreased to 1.98 ± 0.25 kPa, while at deeper indentations (500 nm–1000 nm), the Young’s modulus changed very slightly.

**Figure 4:**
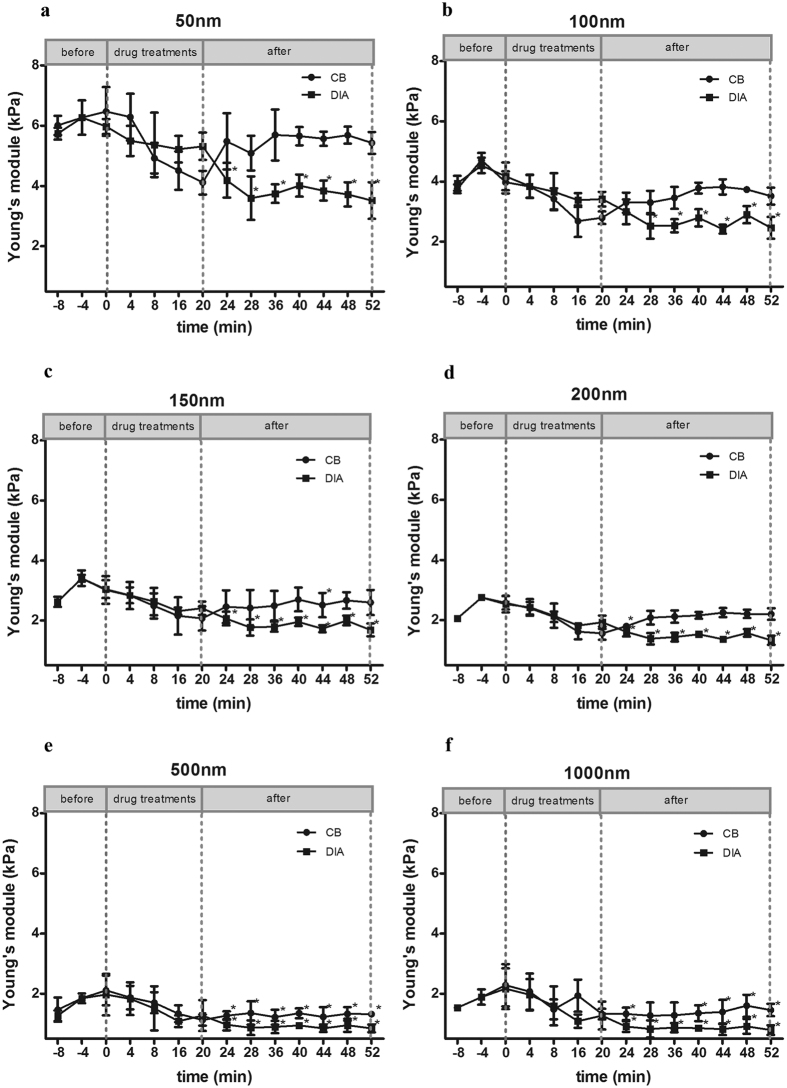
The disruption of the spectrin network or F-actin by DIA or CB both induced a softening effect on osteocytes. At the cortical layer of MLO-Y4 (50–200 nm, **a–d**), the treatment of DIA caused a significant decrease of cell stiffiness after the removal of DIA, but the stiffiness of the CB treated cells were recovered to normal level after the CB removal (**a–d**). At deep layers (500–1000 nm, **e,f**), the stiffiness of MLO-Y4 was decreased significantly after the treatments of both DIA and CB. * Stands for the significant difference of cell stiffiness compared with control (before drug treatments).

The Young’s modulus of MLO-Y4 was measured by AFM. The stiffiness of cell cortex and cell bulk was both focused. The results showed the stiffiness of MLO-Y4 osteocyte was very different from the cortical layer to the deep layer. In the cell cortex (~100 nm phase of the submembrane), the Young’s modulus was 6.31 ± 0.49k Pa, and as the indentation got deeper, to the cell bulk (500 nm), the stiffiness was much smaller with a Young’s modulus of 1.98 ± 0.25 kPa (Fig. 3). The stiffiness of cells after the treatment of DIA or CB was compared. The results showed that the stiffiness of cell cortex (50–200 nm) was not affected by the CB-induced F-actin disruption, while was significantly decreased by the DIA-induced spectrin disruption (Fig. 4a/b/c/d). However, in deep submembrane, at 500 nm and 1000 nm, the cell bulk stiffiness was both decreased by the treatment of CB or DIA (Fig. 4e,f).

### Effect of the spectrin network on intracellular Ca^2+^ signal

**Figure 5:**
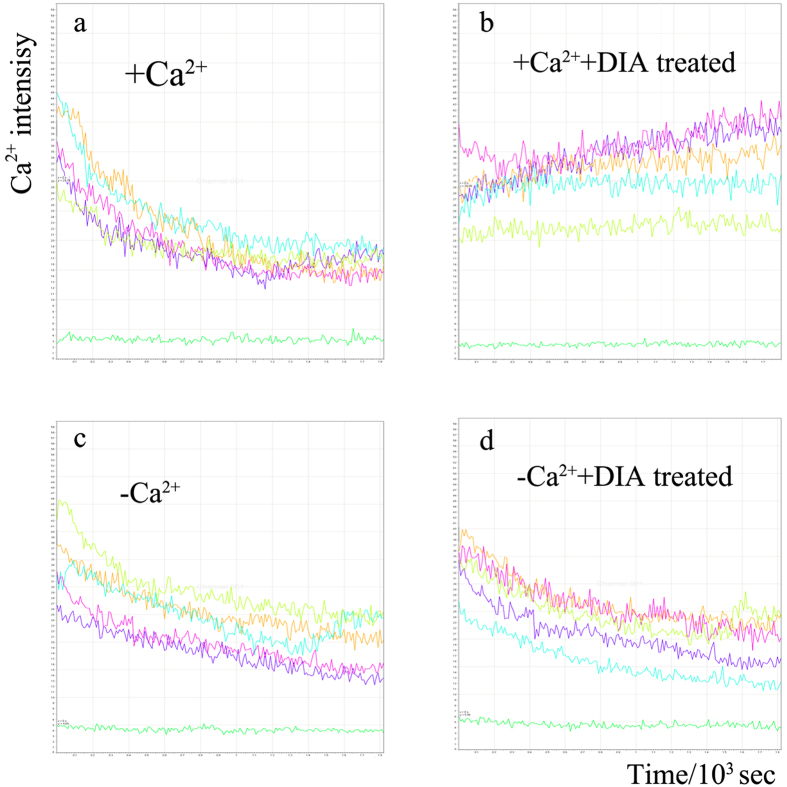
The intracellular Ca^2+^ of MLO-Y4 was fluorescent labeled by Fluo-3 AM and recorded for 30 min after the DIA treatment. In normal medium (+Ca^2+^), the intracellular Ca^2+^ signal attenuated with time owing to the photobleaching effect (**a**), After the DIA treatment, the Ca2+ signal increased although the photobleaching still exist. (**b**) While in the Ca^2+^ free medium (−Ca^2+^), the DIA induced Ca^2+^ increase disappeared and displayed a downward trend along with time (**d**).

The intracellular Ca^2+^ signal of MLO-Y4 was monitored for 30 min after the disruption of spectrin network. The fluorescence of Ca^2+^ signal was recorded as a function of time. Results showed that, the fluorescence intensity of intracellular Ca^2+^ displayed a downward trend with time owing to the photobleaching effect (Fig. 5a). While after the disruption of spectrin network, the intracellular Ca^2+^ signal of MLO-Y4 was significantly increased in normal medium (+Ca^2+^) (Fig. 5b). When the Ca^2+^ source from extracellular was blocked by changing the medium with a calcium-free medium (−Ca^2+^), the Ca^2+^ increase caused by the spectrin disruption was eliminated (Fig. 5c,d).

### Effect of the spectrin network on NO release and eNOS distribution

**Figure 6:**
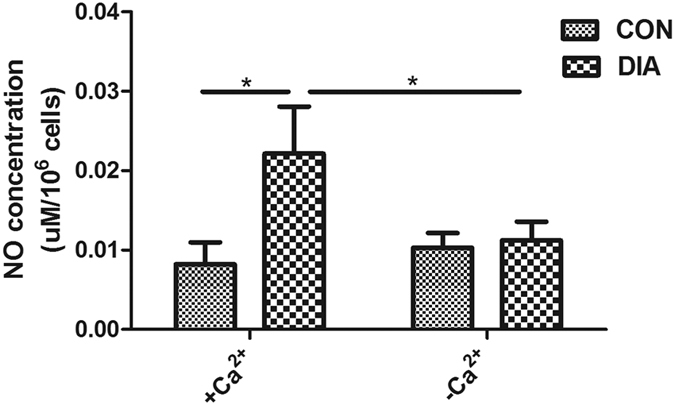
The NO secretion of MLO-Y4 after the DIA treatment in +Ca^2+/^−Ca^2+^ environment. In +Ca^2+^ environment, the disruption of the spectrin network induced a significant increase of NO secretion (175%), while in Ca^2+^ free environment (−Ca^2+^), the DIA-induced NO increase was abrogated. *p < 0.05.

**Figure 7:**
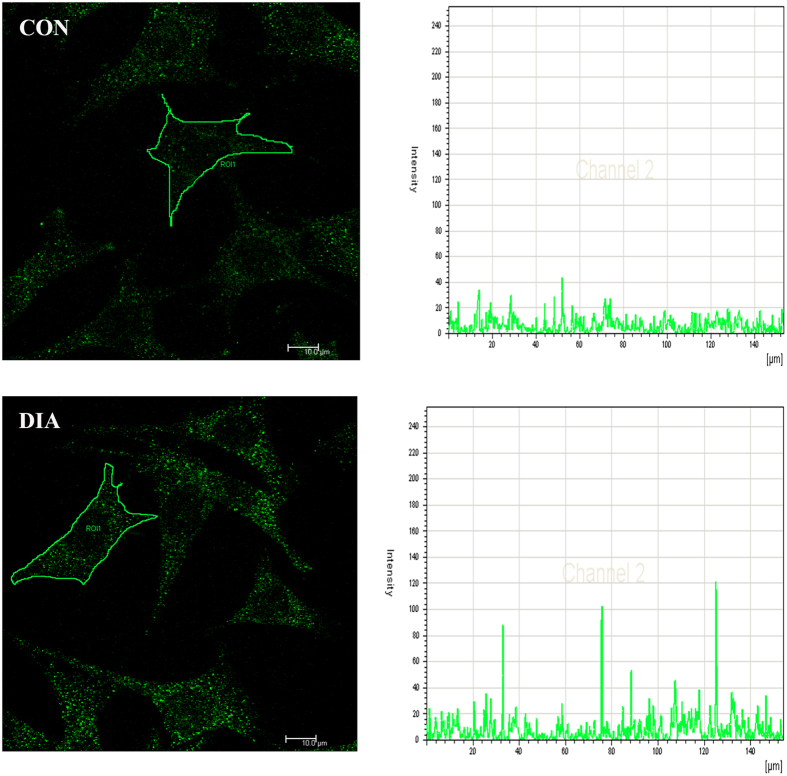
The eNOS of MLO-Y4 osteocyte was fluorescent stained. In the CON group, the eNOS (green) was distributed evenly in the cytoplasm and the intensity profile showed a small variance of the fluorescence distribution. While in the DIA group, the strong staining of the eNOS (indicated by the arrow) and the peak values in the intensity profile both indicated the accumulation of eNOS in the cytoplasm.

The NO secretion and eNOS distribution after the disruption of spectrin network were measured. Results showed that the NO secretion was increased significantly after the DIA treatment. The depletion of extracellular Ca^2+^ did not change the NO secretion, but abrogated the NO increase induced by DIA (Fig. 6). The fluorescence staining showed the eNOS was evenly distributed on the cell membrane and in the cytoplasm in CON. After the disruption of the spectrin network by DIA, the eNOS was aggregated in the cytoplasm, and more intense staining spots can be found in the cytoplasm (Fig. 7, left panel). The outline of a representative cell was drawn and the fluorescence intensity of the inclusive eNOS staining was quantified. The peak values indicated the strong eNOS stainings in MLO-Y4 osteocytes (Fig. 7, right panel).

### Effect of the spectrin network on the formation of cell connections

**Figure 8:**
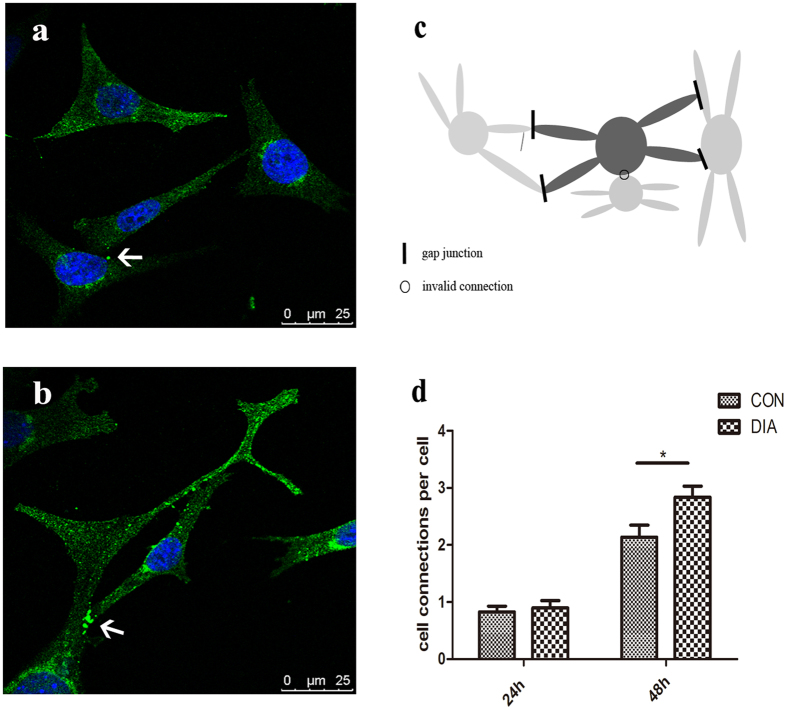
The Cx43 of osteocytes was fluorescent stained and the cell-cell connections of each osteocyte were counted. (**a**) The Cx43 of osteocytes was fluorescent stained (green). In CON group, the Cx43 was distributed both in the cytoplasm and on the cell membrane. Sparse intense staining can be observed at cell-cell contacts. (**b**) After the treatment of DIA, more Cx43 can be found at cell-cell contacts at 48 h after planting. As depicted in (**c**), the connections between cell processes, and the connections between the cell process and cell body were counted, while the connections between the cell bodies were not counted. The cell-cell connections were not fully developed at 24 h after cell planting. At 48 h, the cell-cell connections were formed, and the treatment of DIA promoted the formation of the cell connections by 33% compare with CON (**d**). *p < 0.05.

The gap junction protein connexin 43 (Cx43) of osteocytes was examined by immunofluorescent staining. It was showed that, the Cx43 (green) was distributed both on the cell membrane and in the cytoplasm around the nucleus. Sparse staining of Cx43 can be observed at cell-cell contacts (Fig. 8a, indicated by the arrow). After the treatment of DIA, more intense Cx43 staining between osteocytes processes can be observed (Fig. 8b, indicated by the arrow). The cell-cell connections between osteocytes were also counted based on the schematic of Fig. 8c. The results showed that, the osteocytes did not develop rich connections at 24 h after planting. The average number of cell connections of each osteocyte were less than one. At 48 h after planting, the cell-cell connections were formed. The average number of connections of each osteocyte was 2.14 in CON, and the treatment of DIA significantly increased the formation of the cell-cell connections to 2.84 (Fig. 8d).

## Discussion

In this study, the role of spectrin network in the mechano-sensitive MLO-Y4 osteocytes was explored. It is currently known that the spectrin is organized as a polygonal network in erythrocyte^24^ and a cylindrical lattice in axons^25^. We found that, in MLO-Y4 osteocytes, the spectrin is organized as a porous network, and associates with the F-actin into a dense layer beneath the cell membrane. This spectrin network can provide mechanical support to the cell cortex and cell bulk of MLO-Y4 osteocytes, and is involved in the NO secretion and Ca^2+^ signal pathway, as well as the formation of cell-cell connections of osteocytes.

The Young’s modulus or elastic property of cell is of great importance to the cellular mechano-sensation^17^,^26^. The activation of multiple ion channels and conformation change of some membrane receptors are dependent on the membrane deformation^27^,^28^. In most studies, the Young’s moduli of cells measured by AFM were obtained at 500–1000 nm indentation depth^29^,^30^, where the Young’s modulus is dominated by the cytoskeleton, while the effect of cell cortex is often ignored. In this study, the Young’s moduli of the cell cortex and cell bulk were both focused. The AFM results showed, unlike the F-actin, the disruption of the spectrin-based skeleton caused a holistic softening of MLO-Y4 osteocytes, both in the cell cortex and cell bulk. While the F-actin disruption only caused the stiffiness decrease of cell bulk without affecting the cell cortex. It indicates that the spectrin network is functioned as a platform for the cell cortex, and also a structural scaffold for the cytoplasm, contributing to the cell stiffiness from the upper layer to the deep layer.

The softening of cell cortex will induce the increase of Ca^2+^ channel open probability and eNOS activity, and the role of the cortex F-actin was widely studied before^28^,^31^. Intriguingly, the cell softening induced by spectrin disruption also caused the increase of Ca^2+^ entry and eNOS activity in this study. Wu *et al*.^32^ has demonstrated that the spectrin can selectively control the Ca^2+^ release and Ca^2+^ entry through interaction with protein 4.1, and this finding not only detailed the conformational model^33^ and secretion-like model of Ca^2+^ entry^34^, but also implicated an important role of spectrin in Ca^2+^ entry process. Besides, the spectrin-based membrane skeleton is able to stabilize the ion channels in discrete cellular microdomains^35^, and is essential for the localization of multiple membrane proteins required for Ca^2+^ regulation^36^. Based on above, it is speculated that the disruption of spectrin network possibly increased the mobility and activity of the Ca^2+^ entry channel, and thus induced the Ca^2+^ influx to cytoplasm.

The eNOS, in inactive state, is located in the caveolae on the membrane and ER^37^. The spectrin, together with the dystrophin, is necessary for the caveolae’s restriction to eNOS^38^. When activated, the eNOS will be released from the membrane and transferred to the cytoplasm where it generates NO. It is in consistence with our findings that the disruption of spectrin network caused the accumulation of eNOS in cytoplasm and further induced the dramatic increase of NO production. Moreover, the NO increase induced by spectrin disruption is also Ca^2+^-dependent.

Ursitti has demonstrated that the targeting of Cx43 to the gap junction is relied on αII-spectrin fragments^39^. In this study, the disruption of spectrin network increased the number of gap junction between osteocytes and more connexins were found locating on the cell membrane. So it is speculated that, when the spectrin network is disrupted, more Cx43 will be transported by the dissociative spectrin units to the gap junction.

In addition, the spectrin is also abundantly found in the nucleus of osteocytes (Fig. 1), indicating that the spectrin may also be involved in the nucleoskeleton. The nucleus is recently emerged to be involved in the mechanotransduction of cells^40^,^41^. The nesprin, also called the nuclear envelope spectrin, is demonstrated to be a key element in the nuclear mechanotransduction^42^. Although the function of spectrin in DNA repair in the nucleus has been widely studied^43^,^44^, its role in the nuclear mechanotransduction, especially in the linkage between the cytoskeleton and nucleoskeleton has not been paid much attention to, and further studies need to be performed in the future.

**Figure 9:**
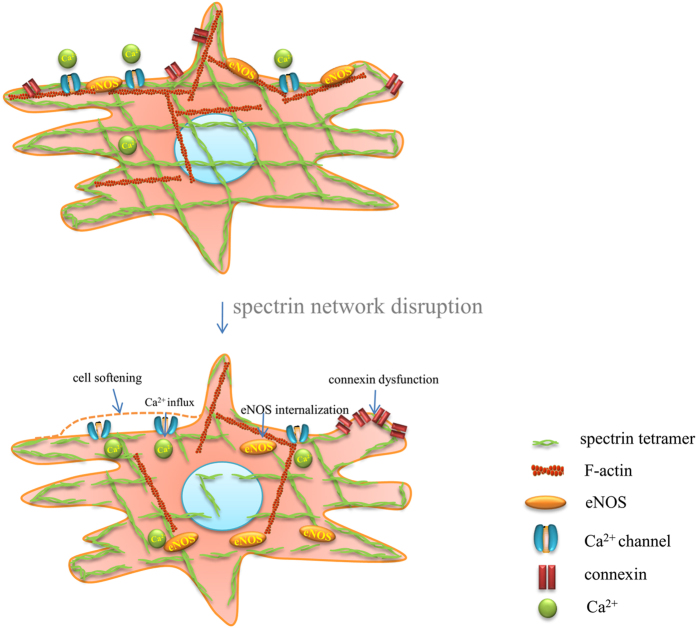
A model for the functions of spectrin in MLO-Y4 osteocyte. The spectrin tetramer is connected with each other and forms a network through the osteocyte. The spectrin network is cross-linked with the F-actin under the plasma membrane. The membrane-associated Ca^2+^ channel, eNOS and connexins were anchored by the spectrin network in the microdomain on the membrane. When disrupted, the anchoring effect of spectrin to Ca^2+^ channel, eNOS and connexins was broken, and caused the influx of Ca^2+^, the translocation of eNOS and the increase of cell-cell connections.

In conclusion, we find that the spectrin network is a global structural support for MLO-Y4 osteocytes, and is essential for the cells’ elasticity maintaining and normal function of the transmembrane Ca^2+^ channel, Cx43 and eNOS (Fig. 9), making it a potential key element in the cellular mechanical-coupled system. A more precise look at the spectrin network structure will provide a detailed description of the mechanism of its function in mechanotransduction of osteocytes.

## Methods

### Cell Culture and treatments

MLO-Y4 cell line (kindly provided by Dr. Lynda Bonewald, University of Missouri-Kansas City, Kansas City, MO) was cultured as described previously^45^. Briefly, cells were maintained in alpha Minimum Essential Medium (α-MEM) (Gibco, USA) supplemented with 5% (vol/vol) fetal bovine serum (FBS) (HyClone, South America) and 5% (vol/vol) calf serum (CS) (HyClone, New Zealand) at 37 °C, 5% CO_2_. The calcium-free MEM (M8020, Sigma-Aldrich, USA) was used to exclude the effect of extracellular Ca^2+^. Culture flasks and culture plates (Nunc, Roskilde, Denmark) pre-coated with rat tail type I collagen (0.15 mg/ml, Millipore, USA) were used for different experiments.

Diazene dicarboxylic acid bis [N,N-dimethylamide] (diamide, DIA, Sigma-Aldrich, MO, USA), a reagent preferentially acts on the spectrin protein was used to increase the link within the spectrin network and disrupt the binding between F-actin and spectrin^46^. Cytoskeleton B (CB, Sigma-Aldrich, MO, USA) was used to depolymerize the F-actin. The DIA and CB were dissolved in DMSO as stock solutions and diluted to 500 uM and 20 uM respectively which were tested to be cytotoxicity-free by MTT assay. After 20 min with the DIA/CB treatment, cells were washed with PBS three times for following experiments.

### Immunofluorescence

MLO-Y4 cells were fixed with 4% paraformaldehyde for 20 min. Primary antibodies (purchased from Santa Cruz, USA) including the mouse monoclonal antibody to spectrin β II (1:200 dilution), mouse monoclonal antibody to Connexin 43 (1:150 dilution) and rabbit polyclonal antibody to eNOS (1:150 dilution) were used. After the incubation with primary antibodies, cells were incubated with the secondary antibodies (purchased from Beyotime, China) including the goat anti-mouse Alexa 488 (1:200 dilution) and donkey anti-rabbit Alexa 488 (1:200 dilution). F-actin was immunofluorescent-stained with phalloidin-rhodamine and the nucleus was labeled with 4′,6-diamidino-2-phenylindole (DAPI) (Invitrogen, USA). Images were captured with a Laser Scanning Confocal Microscope (Leica Microsystems, Wetzlar, Germany), and 40× or 63× oil immersion lens was used.

### Young’s modulus measurement by AFM

An atomic force microscope (5500, Agilent, CA) mounted on an inverted light microscope (TE2000U, Nikon, JPN) was used to measure the Young’s modulus of MLO-Y4 cells. A silicon nitride (SI3N4) tipless tip (TL-CON-20, Nanosensors, CH) was adopted. A silica sphere with diameters ranging from 10 um-20 um was glued by resin cement (SE BOND, Japan) on the tip^47^. The force-distance curves of cells were collected every 4 min. Before the drug treatments, the Young’s modulus of each cell was measured at three time points (−8 min, −4 min, 0 min) for control. After 20 min treatments, the DIA or CB was discarded, and the Young’s modulus was measured for another 32 min (20 min-52 min). DMSO control was also conducted. The object stage was kept fixed to make sure the indentation point on each cell was constant during the experiments. Five force-distance curves were collected by Picoview 1.12 software (Agilent, CA) a t every time point and more than ten cells were measured for each group. The curves were analyzed based on Hertz model for spherical tips^48^ in MATLAB (version 7.0).

### NO measurement

Cells treated with DIA were washed with PBS and cultured in normal medium or calcium-free medium 1 h for NO accumulation. NO^2−^ concentration in the medium was quantified by Griess method by a Nitric Oxide Assay Kit at room temperature in dark environment (Beyotime, China). The standard substance was diluted by normal medium or calcium-free medium respectively. Blank control was set and cell counting was done for normalization.

### Intracellular Ca^2+^ measurement

To visualize the intracellular Ca^2+^ change of cells, Fluo-3 AM (Beyotime Biotechnology, Jiangsu, China), a fluorescence probe for intracellular Ca^2+^ was used. MLO-Y4 cells were washed with PBS for 3 times and incubated in Fluo-3 AM solution (1 uM diluted by normal medium or calcium-free medium) for 30 min at 37 °C. For better de-esterification of Fluo-3 AM, cells were incubated in PBS for another 30 min. After being loaded with Fluo-3 AM, cells were treated by DIA (diluted by normal medium or calcium-free medium). The confocal microscope (Leica Microsystems, Wetzlar, Germany) was used for image capturing (excitation wavelength was 488 nm and the emission wavelength was 530 nm) at the rate of frame/10 s for 30 min. The change of intracellular Ca^2+^ was determined by the fluorescent intensity of Fluo-3 AM and an image processing software (Leica Application Suite) was used for fluorescent intensity measurement.

### Cell connections measurements

MLO-Y4 osteocytes were treated with 500 uM DIA for 20 min at 24 h after planting (2000 cells/cm^2^), and starved in α-MEM for the formation of gap junctions. The connexin protein Cx43 of osteocytes was also immunofluorescent stained and the cell connections were counted at 48 h. The cell connections counting was mainly based on the methodology developed by Wu^49^, and only the connections between processes and the connections between cell process and cell body were counted, while the connections between cell bodies were excluded. Each cell was measured independently. Control group was set and more than 50 cells were measured for each group.

### Statistical analysis

All values were expressed as mean ± standard deviation (SD). Statistical analysis was performed by repeated measurements analysis of variance and unpaired T test. (GraphPad Prism 5.01). When P value was under 0.05, the variance was thought to be significant. All experiments were repeated at least three times.

## Additional Information

**How to cite this article**: Wu, X.-T. *et al*. The potential role of spectrin network in the mechanotransduction of MLO-Y4 osteocytes. *Sci. Rep.* **7**, 40940; doi: 10.1038/srep40940 (2017).

**Publisher's note:** Springer Nature remains neutral with regard to jurisdictional claims in published maps and institutional affiliations.

## Figures and Tables

**Table 1:** Measurement of grids of the spectrin network.

	Diameter (um)	Area (um^2^)	Number of measurements	Number of cells
CON	0.73 ± 0.29	0.35 ± 0.02	510	10
DIA	0.93 ± 0.39^*^	0.67 ± 0.03^*^	468	10
CB	0.85 ± 0.38^*^	0.58 ± 0.05^*^	480	10

*p < 0.05, significant difference with CON.
